# Functional Characterization of a Novel R2R3-MYB Transcription Factor Modulating the Flavonoid Biosynthetic Pathway from *Epimedium sagittatum*

**DOI:** 10.3389/fpls.2017.01274

**Published:** 2017-07-19

**Authors:** Wenjun Huang, Haiyan Lv, Ying Wang

**Affiliations:** ^1^Key Laboratory of Plant Germplasm Enhancement and Specialty Agriculture, Wuhan Botanical Garden, Chinese Academy of Sciences Wuhan, China; ^2^Key Laboratory of South China Agricultural Plant Molecular Analysis and Genetic Improvement, South China Botanical Garden, Chinese Academy of Sciences Guangzhou, China; ^3^Provincial Key Laboratory of Applied Botany, South China Botanical Garden, Chinese Academy of Sciences Guangzhou, China

**Keywords:** *Epimedium*, flavonoid, anthocyanin, flavonol, MYB, transcriptional regulation

## Abstract

*Epimedium* species have been widely used both as traditional Chinese medicinal plants and ornamental perennials. Both flavonols, acting as the major bioactive components (BCs) and anthocyanins, predominantly contributing to the color diversity of *Epimedium* flowers belong to different classes of flavonoids. It is well-acknowledged that flavonoid biosynthetic pathway is predominantly regulated by R2R3-MYB transcription factor (TF) as well as bHLH TF and WD40 protein at the transcriptional level. *MYB* TFs specifically regulating anthocyanin or flavonol biosynthetic pathway have been already isolated and functionally characterized from *Epimedium sagittatum*, but a *R2R3-MYB* TF involved in regulating both these two pathways has not been functionally characterized to date in *Epimedium* plants. In this study, we report the functional characterization of *EsMYB9*, a *R2R3-MYB* TF previously isolated from *E. sagittatum*. The previous study indicated that *EsMYB9* belongs to a small subfamily of *R2R3-MYB* TFs containing grape *VvMYB5a* and *VvMYB5b* TFs, which regulate flavonoid biosynthetic pathway. The present studies show that overexpression of *EsMYB9* in tobacco leads to increased transcript levels of flavonoid pathway genes and increased contents of anthocyanins and flavonols. Yeast two-hybrid assay indicates that the C-terminal region of *EsMYB9* contributes to the autoactivation activity, and *EsMYB9* interacts with *EsTT8* or *AtTT8 bHLH* regulator. Transient reporter assay shows that *EsMYB9* slightly activates the expression of *EsCHS* (chalcone synthase) promoter in transiently transformed leaves of *Nicotiana benthamiana*, but the addition of *AtTT8* or *EsTT8 bHLH* regulator strongly enhances the transcriptional activation of *EsMYB9* against five promoters of the flavonoid pathway genes except *EsFLS* (flavonol synthase). In addition, co-transformation of *EsMYB9* and *EsTT8* in transiently transfected tobacco leaves strongly induces the expressions of flavonoid biosynthetic genes. The potential role of *EsMYB9* in modulating the biosynthesis and accumulation of sucrose-induced anthocyanin and flavonol-derived BCs is also discussed. These findings suggest that *EsMYB9* is a novel *R2R3-MYB* TF, which regulates the flavonoid biosynthetic pathway in *Epimedium*, but distinctly different with the anthocyanin or flavonol-specific *MYB* regulators identified previously in *Epimedium* plants.

## Introduction

*Herba Epimedii*, an important traditional Chinese medicine, is derived from the dried aerial parts of *Epimedium* species in the family Berberidaceae (Huang et al., 2016a). In addition to traditionally being used as a kidney tonic and antirheumatic medicinal herb for more than 2000 years, *Herba Epimedii* has also been currently used for preventing or treating sexual dysfunction, osteoporosis, cardiovascular disease, tumor, and so on Ma et al. (2011) and Jiang et al. (2015, 2016). These significant therapeutic effects on human health are predominantly attributed to the flavonoid compounds, especially C8-prenylated flavonol glycosides, which have been well-confirmed to be the main bioactive components (BCs) in *Epimedium* plants (Ma et al., 2011; Jiang et al., 2015). Additionally, *Epimedium* species are also widely planted as groundcovers and ornamental plants in certain western countries, due to the attractive shape and the diverse color of foliage and flower (Zhang et al., 2014). A wide variety of flower color, ranging from white, yellow, pink, red to purple, is largely due to the accumulation of anthocyanins. Both anthocyanins and flavonol-derived BCs belong to different classes of plant flavonoid metabolites.

As we know, the flavonoid biosynthetic pathway has been extensively and deeply studied in several model plant species, such as *Arabidopsis*, petunia, maize and grape, and almost all the structural genes of this pathway have been identified (Winkel-Shirley, 2001; Koes et al., 2005). In *Epimedium* plants, although the flavonoid-derived BCs have been phytochemically and pharmacologically well-studied previously (Ma et al., 2011; Jiang et al., 2015), the researches about their biosynthesis and regulation at the molecular level started late. In recent years, the flavonoid biosynthetic pathway in *Epimedium sagittatum* has been clarified, and majority of the structural genes of this pathway have been isolated (Zeng et al., 2013; Huang et al., 2015). Moreover, it has been well-acknowledged that the flavonoid biosynthetic pathway is predominantly controlled by *MYB* and *bHLH* transcription factors (TFs), together with WD40 protein at the transcriptional level (Hichri et al., 2011a; Xu et al., 2015). As an increasing number of *MYB* TFs involved in regulating flavonoid biosynthetic pathway have been identified from many plant species, and most of them are key regulators of the synthesis of anthocyanins (Liu et al., 2015). In *Epimedium* plants, three *MYB* regulators, including *EsMYBA1, EsAN2* and *EsMYBF1* have been also isolated and functionally characterized to regulate anthocyanin or flavonol biosynthetic pathway (Huang et al., 2013a, 2016a,b). However, there is another *MYB* gene, *EsMYB9* previously isolated from *E. sagittatum* and suggested to probably regulate the flavonoid biosynthetic pathway (Huang et al., 2013b), but its function has not been clearly characterized until now.

It is well-documented that different R2R3-MYB family members specifically regulate the different branches of flavonoid pathway in both *Arabidopsis* and grape. For example, during the developing grape berries, *VvMYBA1* and *VvMYBA2* regulate anthocyanin synthesis (Kobayashi et al., 2004; Walker et al., 2007), *VvMYBPA1* and *VvMYBPA2* control proanthocyanidin (PA, also known as condensed tannin) synthesis (Bogs et al., 2007; Terrier et al., 2009), and *VvMYBF1* specifically regulates flavonol synthesis (Czemmel et al., 2009). Similarly in *Epimedium* plants, *EsMYBA1* and *EsAN2* TFs regulated anthocyanin synthesis in a tissue-specific manner (Huang et al., 2013a, 2016b), while *EsMYBF1* TF specifically regulated flavonol synthesis (Huang et al., 2016a). However, there is an existing small *MYB* clade, including *VvMYB5a* and *VvMYB5b* from grape, to regulate the whole flavonoid pathway, and their overexpression in tobacco can modulate the biosynthesis and accumulation of the different classes of flavonoids, including anthocyanin, PA, flavonol and lignin (Deluc et al., 2006, 2008). Corresponding in *Epimedium* plants, it was previously reported that *EsMYB9* TF revealed a high level of sequence similarity with *VvMYB5a* and *VvMYB5b* regulators, and also phylogenetically related to the VvMYB5a/b clade (Huang et al., 2013b). Therefore, whether *EsMYB9* has a similar function with *VvMYB5a/b* regulators to regulate the whole flavonoid pathway, especially anthocyanin and flavonol pathways is worthy of being further investigated.

Majority of *MYB* regulators of both anthocyanin and PA biosynthetic pathways generally depend on *bHLH* TF as cofactor (Baudry et al., 2004; Gonzalez et al., 2008), while *MYB* regulator of flavonol biosynthetic pathway can function independently without *bHLH* cofactor (Mehrtens et al., 2005; Czemmel et al., 2009; Huang et al., 2016a). As for the *VvMYB5a* and *VvMYB5b* regulators of the flavonoid biosynthetic pathway, their abilities of interaction with *bHLH* TF have not been described until now. Herein, the relationship analysis of *EsMYB9* and *bHLH* regulator was included in this study. In short, the putative *EsMYB9* regulator of flavonoid biosynthetic pathway was functionally characterized in detail by overexpression in transgenic tobacco, yeast two-hybrid assay, dual-luciferase reporter assay and transient co-transformation in tobacco. The potential role of *EsMYB9* in regulating the biosynthesis and accumulation of sucrose-induced anthocyanin and flavonol-derived BCs in leaves of *Epimedium* was also discussed. Finally, the functional characterization of *EsMYB9* regulator provides new insight into understanding the regulatory mechanisms of both anthocyanin and flavonol-derived BCs biosynthesis in *Epimedium* plants.

## Results

### Overexpression of *EsMYB9* in Tobacco Increases the Accumulation of Anthocyanins and Flavonols

**FIGURE 1:**
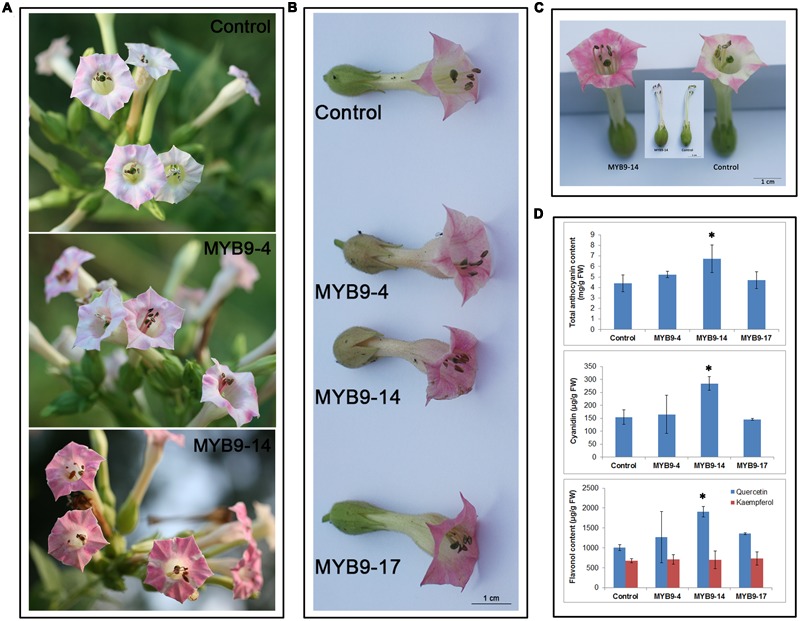
Phenotype observation and flavonoid content measurement in transgenic tobacco overexpressing *EsMYB9* gene. Three representative independent transgenic tobacco lines carrying *EsMYB9* gene (MYB9-4, -14, and -17) and the transgenic tobacco carrying the empty vector as the control plant are indicated. Flowers on plants of the T_1_ transgenic tobacco lines **(A)**, flowers removed from the T_2_ transgenic tobacco lines **(B)**, comparison of flowers of the T_2_ MYB9-14 line with the strongest change of phenotype and control plant **(C)**, and flavonoid content **(D)**, including total anthocyanin, cyanidin, and flavonol in transgenic tobacco flowers are showed. Each column represents mean values with SD bar from three biological replicates for each sample. Asterisk indicates a significant difference from the control plant (*P* < 0.05, LSD test).

To characterize the role of *EsMYB9* TF in regulating the flavonoid biosynthetic pathway, its overexpression in tobacco was carried out. The results of phenotypic changes showed that the red color of flower petals of the transgenic tobacco carrying *EsMYB9* was remarkably enhanced, compared to that of control plants carrying the empty vector (**Figures 1A,B**). In particular, the MYB9-14 overexpression line had a strongest change of flower color. Moreover, the ends of filaments close to anther tissue from the overexpression lines turned from pale green to pink (**Figure 1C**). In addition, the whole filaments of flowers from the T_1_ overexpression lines turned to pink (data not shown). This enhancement of red pigments was probably attributed to the increasing accumulation of anthocyanins. Therefore, the contents of flavonoids, including anthocyanins and flavonols in transgenic tobacco flowers were determined. The results indicated that the contents of total anthocyanin, anthocyanin cyanidin, flavonol (especially quercetin) were significantly increased in the MYB9-14 overexpression line, compared to the control (**Figure 1D**). As for other two overexpression lines (MYB9-4 and MYB9-17), only the flavonol quercetin content was moderately higher than that of control, but other flavonoids were not significantly increased (**Figure 1D**). Finally, these results suggested that overexpression of *EsMYB9* in tobacco can lead to increase the accumulation of anthocyanins and flavonols in flowers.

### Overexpression of *EsMYB9* in Tobacco Influences the Expression Levels of Flavonoid Pathway Genes

**FIGURE 2:**
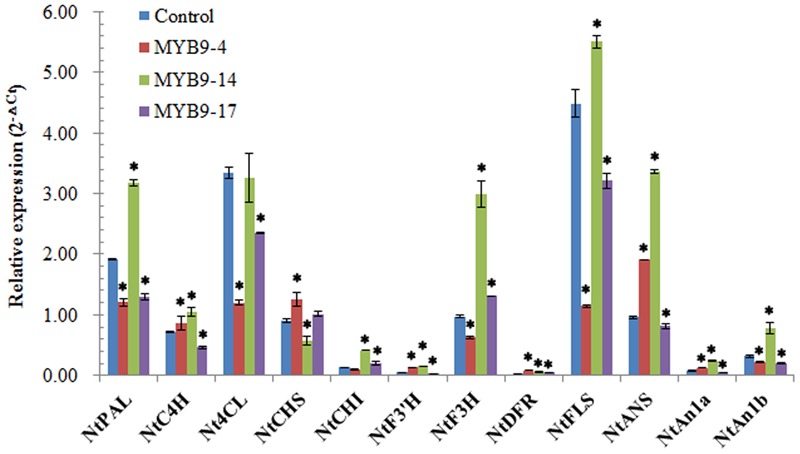
Quantitative PCR assay of the flavonoid pathway genes in transgenic tobacco flowers carrying *EsMYB9* gene. The expression levels of 10 structural genes of flavonoid biosynthetic pathway and two *bHLH* regulatory genes (*NtAn1a* and *NtAn1b*) are determined by quantitative PCR assay. Tobacco *Tub1* gene is used as an internal control, and comparative Ct method is used to determine the relative expression level. Three *EsMYB9* overexpressing tobacco lines (MYB9-4, -14, and -17) and the control plant carrying the empty vector are used for qPCR assay. Each column represents average value with SD bar from three technical replicates. Asterisk represents a significant difference from the control plant at the level of *P* < 0.05 using LSD statistical analysis.

The impact of *EsMYB9* overexpression on the transcript levels of flavonoid pathway genes in transgenic tobacco flowers was analyzed by qPCR assay. The presence of introduced *EsMYB9* in transgenic tobacco was firstly verified by semi-quantitative RT-PCR assay. The results indicated that *EsMYB9* was most abundantly expressed in the MYB9-14 overexpression line, followed by in the MYB9-4 overexpression line, and lowly expressed in the MYB9-17 overexpression line (**Supplementary Figure S1**). Secondly, the qPCR results showed that most of anthocyanin biosynthetic genes and two *bHLH* regulatory genes, including *NtCHI* (chalcone isomerase), *NtF3*′*H* (flavonoid 3′-hydroxylase), *NtF3H* (flavanone 3-hydroxylase), *NtDFR* (dihydroflavonol 4-reductase) and *NtANS* (anthocyanidin synthase) as well as *NtAn1a* and *NtAn1b* were significantly up-regulated in two overexpression lines (MYB9-4 and MYB9-14), especially in the MYB9-14 line, compared to the control (**Figure 2**). However, only in the MYB9-14 overexpression line, the expression level of *NtFLS* was slightly and significantly higher than that of the control (**Figure 2**). In addition, the transcript levels of almost all the general phenylpropanoid pathway genes, including *NtPAL* (phenylalanine ammonia-lyase), *NtC4H* (cinnamate 4-hydroxylase) and *Nt4CL* (4-coumaroyl:CoA-ligase) were not considerably changed by ectopic expression of *EsMYB9*, and they all were abundantly expressed in three overexpression lines and control plant (**Figure 2**). In the MYB9-17 overexpression lines carrying the lowest expression level of *EsMYB9*, the change extent of gene expression level was obviously lower than in other two overexpression lines (**Figure 2**). Finally, these results indicated that overexpression of *EsMYB9* in tobacco strongly promoted the expression levels of main anthocyanin pathway genes and two *bHLH* regulatory genes, and the effect on the *NtFLS* expression level have to be confirmed further.

### *R2R3-MYB* Regulator *EsMYB9* Interacts with *bHLH* Regulators

**FIGURE 3:**
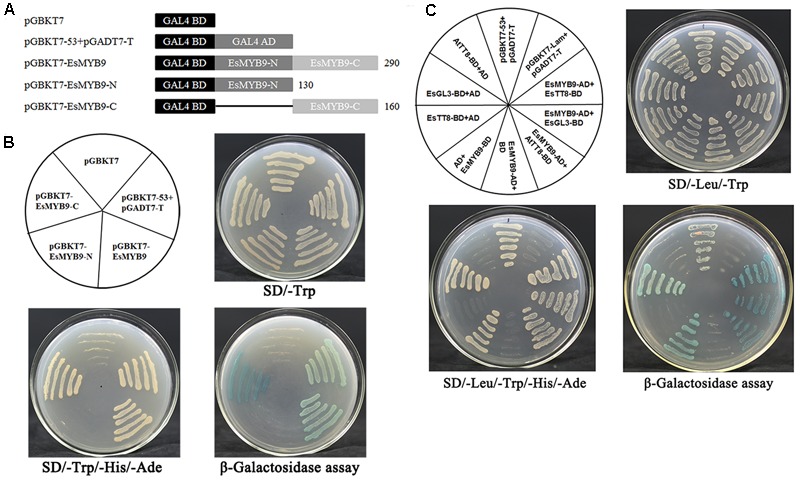
Yeast two-hybrid assay of EsMYB9 transcription factor (TF) with three bHLH regulators of the flavonoid pathway. Three different regions of EsMYB9 protein are used for transcription autoactivation assay, including the full-length coding region, the R2R3 MYB domain-containing N-terminal region and the C-terminal region **(A)**. Transcription autoactivation assay **(B)** of EsMYB9 and its interaction with bHLH proteins **(C)** by yeast two-hybrid assay are indicated. Three bHLH proteins involved in regulation of the flavonoid biosynthetic pathway, EsTT8 and EsGL3 from *Epimedium sagittatum* and AtTT8 from *Arabidopsis thaliana* are used for yeast two-hybrid assay. Transformed yeast cells harboring pGBKT7-53 + pGADT7-T, or pGBKT7-Lam + pGADT7-T constructs are used as a positive and a negative control, respectively.

Considering the presence of the conserved bHLH interacting motif in EsMYB9 protein and the up-regulation of *NtAn1a* and *NtAn1b bHLH* regulators by ectopic expression of *EsMYB9* in tobacco, yeast two-hybrid assay (Y2H) was carried out to detect the interaction of EsMYB9 with bHLH regulators. Autoactivation activity of EsMYB9 was firstly detected using three regions of EsMYB9 protein, including the coding region, the N-terminal and C-terminal region (**Figure 3A**). The results showed that the whole EsMYB9 protein possessed a strong activity of autoactivation, and the C-terminal revealed a stronger autoactivation activity but the N-terminal did not (**Figure 3B**), suggesting that the autoactivation activity of EsMYB9 TF must be attributed to the C-terminal region. For interaction analysis of EsMYB9 with bHLH proteins in Y2H assay, three bHLH regulatory proteins of the flavonoid pathway, including EsTT8, EsGL3 from *E. sagittatum* and AtTT8 from *Arabidopsis thaliana* were selected. The results showed that the transformed yeast cells harboring EsMYB9-AD and EsTT8-BD, or EsGL3-BD or AtTT8-BD construct combinations were successfully passed through the double and quadruple selection plates, and the blue colonies occurred in the corresponding yeast cells through β-galactosidase assay (**Figure 3C**). Meanwhile, the yeast cells transformed with EsTT8-BD or AtTT8-BD plus with the empty AD vector combination did not grow normally on the quadruple and the β-galactosidase selection plates, but the combination of EsGL3-BD and AD constructs passed through these selections (**Figure 3C**), indicating that EsGL3 possessed an activity of autoactivation, but EsTT8 and AtTT8 did not. In addition, both the positive and negative controls were as expected. Finally, the Y2H results indicated that EsMYB9 can interact with EsTT8 or AtTT8 bHLH regulators, but the interaction with EsGL3 can be excluded due to the presence of the autoactivation activity of EsGL3 protein found.

### *BHLH* Is Required for Promoting the Transcriptional Activation of *EsMYB9* against Promoters of the Flavonoid Pathway Genes

**FIGURE 4:**
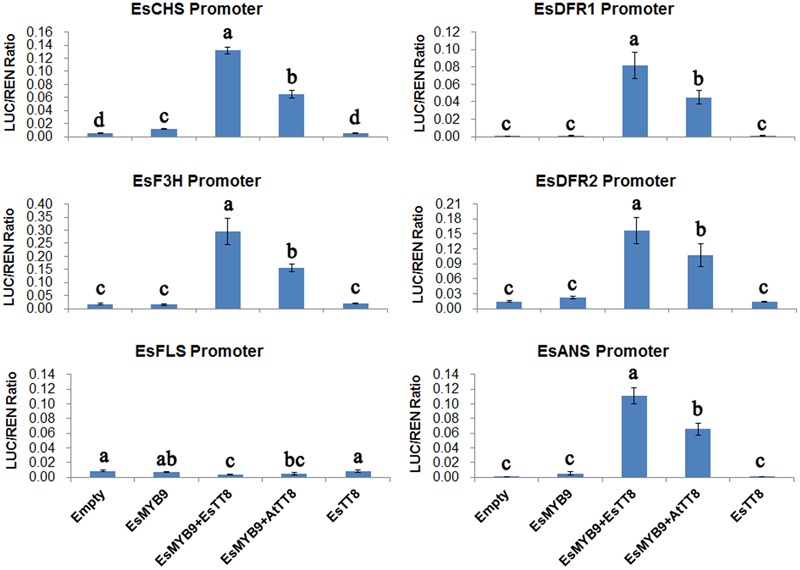
Transcription activation assay of *EsMYB9* against promoters of the flavonoid pathway genes with or without *bHLH* regulators in transiently transformed leaves of *Nicotiana benthamiana*. A total of six promoters of the flavonoid biosynthetic genes from *E. sagittatum* are used for dual-luciferase reporter assay, including *EsCHS, EsDFR1, EsDFR2, EsF3H, EsFLS*, and *EsANS* promoters. Two *bHLH* regulators of the flavonoid pathway, *EsTT8* from *E. sagittatum* and *AtTT8* from *A. thaliana* are also used as cofactors for *EsMYB9*. Transformed leaves carrying only the promoter-LUC reporter construct without the TF-containing effector construct are used as the controls (empty). The columns represent average values with SD bar from at least four biological replicates for each treatment. Values followed by the same letter are not significantly different at the level of *P* < 0.05 (Duncan multiple range test).

Dual-luciferase reporter assay was performed in transiently transformed leaves of *Nicotiana benthamiana* to analyze the transcriptional activation of *EsMYB9* TF against promoters of the flavonoid pathway genes from *E. sagittatum* with/without *EsTT8* or *AtTT8 bHLH* regulator as a cofactor. The results showed that the LUC/REN ratios of promoters of *EsCHS, EsDFR2*, and *EsANS* were increased by approximately 2 to 13-fold when *EsMYB9* was used alone, but only *EsCHS* promoter activity was significantly enhanced, compared to their corresponding empty controls which only contained promoters without any *MYB* or *bHLH* TFs (**Figure 4**). In addition, the activities of *EsDFR1, EsF3H* and *EsFLS* promoters were not significantly changed by *EsMYB9* used alone (**Figure 4**). It is noted that all the LUC/REN ratios of these six promoters treated with either the empty control or *EsMYB9* alone were extremely low. However, the LUC/REN ratios of all these promoters except *EsFLS* were considerably increased when *EsMYB9* with *EsTT8* or *AtTT8 bHLH* regulators were co-transformed, while *EsTT8* alone did not significantly promote the transcriptional activities of these promoters (**Figure 4**). Additionally, any treatment, including either *EsMYB9* alone or its combination with *bHLH* regulators did not largely change the transcriptional activity of *EsFLS* promoter, compared to the empty control (**Figure 4**). In summary, these results indicated that *EsMYB9* was very likely to slightly activate the *EsCHS* promoter, but the addition of *EsTT8* or *AtTT8 bHLH* TFs strongly promoted the transcriptional activation of *EsMYB9* against promoters of the flavonoid pathway genes except *EsFLS*, suggesting that the *bHLH* regulatory protein was required as a cofactor to enhance the transcription activation ability of *EsMYB9* on target promoters in the heterologous system of *N. benthamiana*.

### Co-transformation of *EsMYB9* and *EsTT8* Strongly Induce the Expressions of Anthocyanin Biosynthetic Genes in Transiently Transformed Tobacco Leaves

**FIGURE 5:**
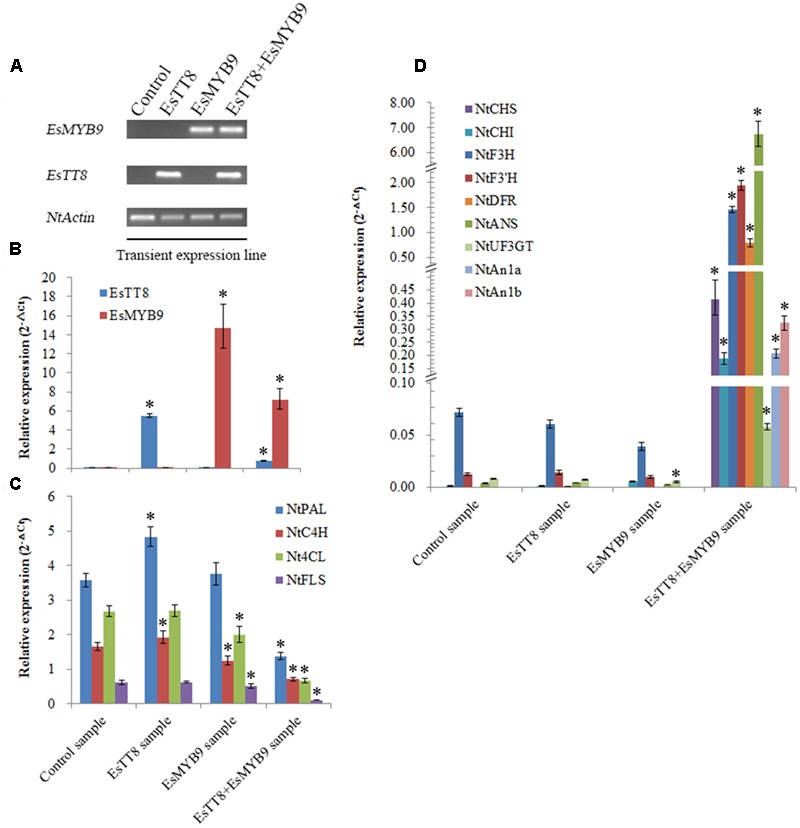
Quantitative PCR assay of the flavonoid pathway genes in tobacco leaves transiently transfected by *EsMYB9* with or without *EsTT8* bHLH regulator. The leaves transiently transfected with the empty vector is used as the control sample, and transiently transfected with *EsTT8* alone or *EsMYB9* alone or their combination is referred to the EsTT8 sample, EsMYB9 sample and EsTT8+EsMYB9 sample, respectively. The presence of introduced *EsMYB9* and *EsTT8* genes in transiently transfected samples is confirmed by RT-PCR assay **(A)**. The strong expressions of EsTT8 and EsMYB9 in transfected samples by their transient expressions **(B)**, the down-regulation of four genes in transfected samples by EsTT8 or/and EsMYB9 transient expressions **(C)** and the remarkable up-regulation of nine genes in transiently co-transformed sample with EsTT8 and EsMYB9 **(D)** are indicated through quantitative PCR assay. Each column represents average value with SD bar from three technical replicates. Asterisk represents a significant difference from the control sample at the level of *P* < 0.05 using LSD test.

In order to further confirm the requirement of *bHLH* regulator as cofactor for *EsMYB9*, the transient expression of *EsMYB9* with or without *EsTT8* regulator was carried out in tobacco leaves. Firstly, the presence of *EsMYB9* and *EsTT8* in the corresponding transiently transformed tobacco leaves were confirmed by RT-PCR assay (**Figure 5A**). Secondly, quantitative PCR assay was performed to monitor the changes of the transcript levels of the flavonoid pathway genes. The results showed that both *EsMYB9* and *EsTT8* were abundantly expressed in tobacco leaves carrying itself alone or their combination (**Figure 5B**). Four structural genes, including *NtPAL, NtC4H, Nt4CL*, and *NtFLS* were almost significantly down-regulated in all three treated samples, particularly in EsTT8 + EsMYB9 co-transformed sample, compared to the control sample transiently transformed by the empty vector (**Figure 5C**). The most striking change is that most of anthocyanin biosynthetic genes, including from *NtCHS* to *NtUF3GT* (UDP-glucose:flavonoid 3-*O*-glucosyltransferase) as well as *NtAn1a* and *NtAn1b bHLH* regulators were remarkably up-regulated in EsTT8+EsMYB9 co-transformed sample (**Figure 5D**). Meanwhile, these anthocyanin biosynthetic genes were not significantly changed by transient expression of either *EsMYB9* or *EsTT8* alone except *NtUF3GT* in EsMYB9 sample (**Figure 5D**). It is worthy to notice that the expression levels of the phenylpropanoid pathway gene, *NtPAL, NtC4H*, and *Nt4CL* were high in the four samples, but all the anthocyanin pathway genes were very lowly expressed in EsTT8 sample and EsMYB9 sample, closely to their background levels in the control sample (**Figures 5C,D**). However, surprisedly, no obvious red pigments occurred in transfected tobacco leaves carrying *EsMYB9* or even with *EsTT8* combination under an optical microscope (data not shown), implying that the anthocyanin accumulation may not be effectively induced by the transient expression of *EsMYB9* with or without *EsTT8* regulator. At least, it is well-confirmed that the co-transformation of *EsMYB9* and *EsTT8* can strongly induce the anthocyanin pathway genes in transiently transformed tobacco leaves.

## Discussion

It was previously reported that apart from the very well-conserved R2R3 MYB domain, three distinct conserved motifs were also found in EsMYB9 protein sequence, including the bHLH interaction motif, the C1 motif initially identified in the R2R3-MYB subgroup 4 proteins and the C3 motif specifically found in the all members of the VvMYB5a/b cluster (Huang et al., 2013b). Moreover, the high degree of sequence similarity and the close phylogenetic relationship of *EsMYB9* with the members of the small VvMYB5a/b cluster may indicate similarity in function. First of all, overexpression of *EsMYB9* in tobacco resulted in a similar phenotypic changes with the reports of *VvMYB5a* and *VvMYB5b* overexpression in tobacco. Pigmentation was clearly increased in petal and filament of transgenic tobacco flower overexpressing *EsMYB9* (**Figure 1**), while flowers of transgenic tobacco overexpressing *VvMYB5a* or *VvMYB5b* showed a strongly increased pigmentation in both petal and stamen tissues (Deluc et al., 2006, 2008). However, it is noticed that the phenotypic changes were only observed in reproductive tissues, not in vegetative tissues of the *EsMYB9* overexpression plants, which agrees with the reports of both *VvMYB5a* and *VvMYB5b* regulators. By contrast, overexpression of certain *MYB* regulators in tobacco induced a strongly increased pigmentation in both reproductive and vegetative tissues, such as *EsMYBA1* and *EsAN2* from *E. sagittatum* (Huang et al., 2013a, 2016b), *AtPAP1* from *A*. *thaliana* (Borevitz et al., 2000) and *NtAN2* from tobacco (Pattanaik et al., 2010), and all these *MYB* regulators have been identified to specifically regulate anthocyanin biosynthesis. Secondly, both anthocyanin and flavonol contents were significantly enhanced in transgenic flowers of the MYB9-14 overexpression line (**Figure 1**). It was previously reported that *VvMYB5a* overexpression increased the biosynthesis of anthocyanins, flavonols and condensed tannins and altered lignin metabolism in tobacco flowers (Deluc et al., 2006), while *VvMYB5b* overexpression resulted in a strong accumulation of anthocyanins and PAs in tobacco flowers (Deluc et al., 2008). These results indicated that the phenotypic change due to *EsMYB9* overexpression was basically similar with that of *VvMYB5a* and *VvMYB5b*, but appeared different with those *MYB* regulators of anthocyanin pathway.

The change of flavonoid content in transgenic tobacco flower implied that the expressions of flavonoid biosynthetic genes must be regulated by *EsMYB9* overexpression. Corresponding to the increased anthocyanin in tobacco flowers expressing *EsMYB9*, most of anthocyanin pathway genes were remarkably up-regulated, including *NtCHI, NtF3′H, NtF3H, NtDFR*, and *NtANS* (**Figure 2**). Similarly, almost all the key anthocyanin structural genes, including *NtCHS, NtCHI, NtF3H, NtDFR*, and *NtANS* were strongly up-regulated in the *VvMYB5a* or *VvMYB5b* overexpressing stamens and petals (Deluc et al., 2006, 2008). Additionally, the expression level of *NtFLS* was significantly decreased in two *EsMYB9* overexpression lines and significantly increased only in the MYB9-14 line (**Figure 2**), which did not well-correspond to the increased flavonol in transgenic flowers. Although the flavonol content was slightly enhanced in transgenic tobacco flowers expressing *VvMYB5a*, the transcript level of *NtFLS* was still not investigated (Deluc et al., 2006). For the general phenylpropanoid biosynthetic genes, including *NtPAL, NtC4H* and *Nt4CL*, their expressions were not considerably changed in the *EsMYB9* overexpression lines compared to the control, although both *NtPAL* and *NtC4H* were clearly increased in one of three *EsMYB9* overexpression lines (**Figure 2**). Further in tobacco leaves transiently transformed by *EsMYB9*, they were also not changed largely (**Figure 5C**). These results are partly consistent with the findings of *VvMYB5a* that the expressions of *NtPAL* and *Nt4CL* appeared unaffected, and *NtC4H* was slightly induced by *VvMYB5a* overexpression in transgenic tobacco leaves (Deluc et al., 2006). Finally, these results indicated that overexpression of *EsMYB9* in tobacco mainly promoted the expressions of anthocyanin biosynthetic genes.

Corresponding to the presence of bHLH interaction motif in EsMYB9 protein sequence, the interaction of *EsMYB9* and *EsTT8* or *AtTT8* bHLH regulators was confirmed by Y2H and dual-luciferase reporter assay (**Figures 3, 4**). To our knowledge, although Y2H assay of *VvMYB5a* or *VvMYB5b* with *bHLH* regulators have not been still investigated, but their interaction was demonstrated by a transient reporter assay (Deluc et al., 2008; Hichri et al., 2011b). As we know, majority of *MYB* regulators of anthocyanin and PA biosynthetic pathways generally interact with *bHLH* regulators as a cofactor to modulate the transcriptional expressions of flavonoid pathway genes, such as *Epimedium EsMYBA1* and *EsAN2* (Huang et al., 2013a, 2016b), and tobacco *NtAN2* regulators (Pattanaik et al., 2010) of anthocyanin pathway, or *Arabidopsis AtTT2* (Baudry et al., 2004), grape *VvMYBPA1, VvMYBPA2, VvMYBPAR* regulators (Bogs et al., 2007; Koyama et al., 2014) for PA pathway. Meanwhile, *MYB* regulators of flavonol pathway function independently without bHLH regulators, including *AtMYB12* from *A. thaliana* (Mehrtens et al., 2005), *VvMYBF1* from grape (Czemmel et al., 2009) and *EsMYBF1* from *E. sagittatum* (Huang et al., 2016a). Moreover, the addition of *EsTT8* or *AtTT8* bHLH regulators strongly enhanced the transcription activation of *EsMYB9* against the promoters of flavonoid biosynthetic genes except *EsFLS* promoter in a transient reporter assay, while *EsMYB9* used alone just slightly activate the expression of *EsCHS* promoter (**Figure 4**). These results suggested that the presence of a bHLH protein was very critical, or even required for *EsMYB9* to activate the expressions of target genes. The considerable up-regulation of the transcript levels of anthocyanin biosynthetic genes by co-transformation of *EsMYB9* with *EsTT8* in a transiently transformed tobacco leaves also support this conclusion (**Figure 5**). A similar result was also found for the activation assay of flavonoid gene promoters by *VvMYB5a, VvMYB5b, VvMYBPA1*, or *VvMYBPAR* regulators in a transient reporter assay (Deluc et al., 2008; Hichri et al., 2011b; Koyama et al., 2014). They can’t significantly and largely induce promoter activities without the expression of *AtEGL3* or *VvMYC1* bHLH regulator, and the addition of these *bHLH* regulators resulted in a remarkably higher level of transcription activity of target promoter than that of these *MYB* regulators used alone. Surprisedly, the expression of *EsFLS* promoter appeared unaffected by expression of *EsMYB9* with or without *EsTT8* (**Figure 4**). This is probably because that an only 624 bp upstream region from ATG start site of *EsFLS* used for the reporter construction is very short, and the potential binding site for *EsMYB9* is absent in this promoter sequence. A longer promoter sequence of *EsFLS* may be needed to further detect its activity by *EsMYB9* regulator.

A coordination relationship was previously suggested between anthocyanin pathway and flavonol-derived BCs pathway during the leaf developmental stage of *E. sagittatum* (Huang et al., 2015). As we know, sucrose treatment can modulate expressions of phenylpropanoid pathway genes and affect biosynthesis and accumulation of phenylpropanoids (Payyavula et al., 2013). For example in *Arabidopsis*, sucrose treatment can strongly induce accumulation of anthocyanins in seedlings through induction of *AtPAP1* regulator (Teng et al., 2005). Therefore, the sterile cultured shoots of *E. sagittatum* were treated with 3 or 5% concentration of sucrose to dissect the potential role of *EsMYB9* in modulating both anthocyanin and flavonol-derived BCs synthesis. The results showed that the contents of the main BCs, epimedin C and total flavonoid were significantly induced in response to sucrose treatment (**Supplementary Figure S2A**). In addition, red pigments were obviously observed in petioles and rhizomes 8 days after sucrose treatment. Corresponding, the expressions of flavonoid biosynthetic genes, including *EsFLS, EsPT2*, and *EsPT3* for synthesis of the flavonol-derived BCs, and *EsDFR1* and *EsANS* for anthocyanin biosynthesis were significantly up-regulated by sucrose treatment (**Supplementary Figure S2B**). Moreover, both *EsMYBF1* and *EsMYBA1* expressions were also significantly up-regulated, especially *EsMYBF1*, which are responsible for the regulation of flavonol pathway and anthocyanin pathway, respectively (**Supplementary Figure S2C**). However, *EsMYB9* expression appeared unaffected by sucrose treatment, as well as *EsAN2* and *EsTT8* regulators (**Supplementary Figure S2C**). These results indicated that sucrose can induce both flavonol and anthocyanin synthesis and accumulation through inducing expressions of the biosynthetic genes of flavonol and anthocyanin pathways and their regulatory genes, mainly including *EsMYBF1* and *EsMYBA1*, but not *EsMYB9*. Hence, *EsMYB9* regulator appeared not involved in the modulation of sucrose-induced anthocyanin and flavonol biosynthesis. In addition, the fact that *EsTT8 bHLH* regulator was not induced may partly contribute to the implementation of *EsMYB9* function.

## Conclusion

A novel *R2R3-MYB* TF, *EsMYB9* isolated from *E. sagittatum* was previously suggested to regulate the flavonoid biosynthetic pathway. Here, this study indicates that overexpression of *EsMYB9* in tobacco results in increased contents of both anthocyanin and flavonol in reproductive tissue through modulating the expressions of flavonoid pathway genes. *EsMYB9* can interact with *EsTT8* or *AtTT8* bHLH regulators and these bHLH proteins are very critical for *EsMYB9* transcription activation ability against target promoters. In addition, co-transformation of *EsMYB9* and *EsTT8* in transiently transfected tobacco leaves strongly induces the expressions of the main flavonoid biosynthetic genes except *FLS* gene. It is also demonstrated that *EsMYB9* is not involved in the regulation of sucrose-induced anthocyanin and flavonol-derived BCs biosynthesis and accumulation in leaves of *Epimedium*. The functional characterization of *EsMYB9* provides new insight into understanding the regulatory mechanism of the biosynthesis of both anthocyanin and flavonol-derived BCs in *Epimedium* plants.

## Materials and Methods

### Plant Materials

Plants of *Nicotiana tabacum* cv. NC89 were used for stable and transient genetic transformation, and *N. benthamiana* was used for dual-luciferase reporter assay. Both they were grown in a greenhouse at 22–25°C with 14 h/10 h of light/dark period until required.

### Construction of Overexpression Vector and Tobacco Transformation

For overexpression of *EsMYB9* in tobacco, the coding region of *EsMYB9* was transferred from pMD19-T cloning vector (Takara, Japan) cut with *Sal* I and *Kpn* I restriction enzymes to the modified binary pMV vector (derived from pBI121 vector) cut with *Xho* I and *Kpn* I restriction enzymes, producing the pMV-EsMYB9 overexpression construct. The *EsMYB9* expression was triggered under the control of CaMV 35S promoter in this construct. This overexpression construct was confirmed by sequencing and then introduced into *Agrobacterium tumefaciens* EHA105 strain by electroporation method for tobacco stable genetic transformation. The leaf disk method described previously (Horsch et al., 1985) was used for *Agrobacterium*-mediated genetic transformation of tobacco. The positive transformed tobacco plants were screened using kanamycin as a plant selective marker and the existence of introduced *EsMYB9* gene in transformants was confirmed by PCR assay. Finally, three representative independent T_2_ transgenic tobacco lines (MYB9-4, -14, and -17) showing the strong, moderate and weak changes of flower colors were used for further analysis. In addition, the transient expressions of *EsMYB9* or *EsTT8* or their combination in tobacco leaves were also carried out as described for the *Agrobacterium*-infiltrated transformation of *N. benthamiana* (Huang et al., 2016a). The detailed protocol was seen in the method of dual-luciferase reporter assay below. The overexpression construct pMV-EsTT8 harboring the full-length coding region of *EsTT8* was used for the transient expression. For the transient co-transformation of *EsMYB9* and *EsTT8*, their suspension culture were equally mixed as the infiltration solution. The infiltrated pots of leaves were harvested for qPCR assay after 3 days of inoculation. Transgenic tobacco expressing the pMV empty vector was used as a negative control in either stable transformation or transient transformation. Primers for the overexpression vector constructions of *EsMYB9* and *EsTT8* were listed in Supplementary Table S1.

### Measurement of Flavonoid Content in Transgenic Tobacco Flowers

To clarify the effect of *EsMYB9* overexpression on flavonoid synthesis and accumulation in transgenic tobacco flowers, the flavonoid extraction and content measurement were performed as described previously (Huang et al., 2015). Total anthocyanin content and flavonoid content were measured using a spectrophotometric method and a HPLC (high performance liquid chromatography) method, respectively. Briefly, the buffer of 1% HCl/methanol was used to extract anthocyanin from tobacco flowers and the absorbance of supernatant was monitored at 530 and 657 nm. The formula A530-0.25^∗^A657 was adopted to compensate for the absorption of chlorophyll degradation products at 530 nm. Total anthocyanin content was determined using a cyanidin-3-*O*-glucoside as a standard. Meanwhile, flavonols were extracted by 80% methanol and sonication. Both anthocyanin and flavonol contents were presented as aglycones by preparing acid-hydrolyzed extracts. Before injection, a 0.22 μm filter membrane was used to filter the hydrolyzation solution. The chromatographic analysis was run on an Agilent 1100 series HPLC system accompanied with an Agilent TC-C18 column (5 μm, 4.6 mm × 250 mm). The mobile phase was comprised of solvent A (0.1% formic acid in water), solvent B (acetonitrile) and solvent C (methanol). The gradient elution program was as follows: initiate from 10% B + 2% C at 0 min; increase to 20% B + 4% C at 10 min; proceed up to 50% B + 10% C at 15 min; drop to 20% B + 4% C at 20 min; back to the initial at 25 min and re-equilibrate for 3 min. The detection wavelength for kaempferol and quercetin flavonols, and cyanidin anthocyanin was set at 350 and 530 nm, respectively. The identification and quantification of flavonoid compounds in tobacco flowers were compared with the commercial standards of kaempferol and quercetin, and cyanidin. The measurement of flavonoid content for each sample was repeated two times using three independent biological replicates.

### Quantitative RT-PCR Assay

qRT-PCR assay was carried out to determine the gene expression level according to Huang et al. (2013a). In brief, total RNA was isolated from flowers and leaves of tobacco using RNAiso plus reagent (Takara, Japan) and its quality and quantity were determined by electrophoresis and NanoDrop 2000 spectrophotometer (Thermo Scientific, United States). Before reverse transcription, gDNA eraser (Takara, Japan) was used to digest total RNA for removing any contaminated genomic DNA. Then, the digested RNA solution was reversely transcribed by PrimeScript RT reagent kit (Takara, Japan) following the supplier’s recommendation. Finally, the qPCR assay was set up with SYBR Premix Ex Taq II (Tli RNase H Plus) kit (Takara, Japan) and performed in an ABI7500 Fast Real-Time PCR equipment (Applied Biosystems, United States) following the manufacturer’s instruction. The qPCR program was as follows: 95°C for 30 s, 40 cycles of 95°C for 3 s and 60°C for 30 s, and a default melt curve program. The comparative Ct method was used to analyze the gene expression level (Schmittgen and Livak, 2008). Tobacco *NtTub1* genes was used as an internal control of qPCR assay. Primers used for qPCR assay of the flavonoid pathway genes in tobacco were listed in Supplementary Table S2.

### Yeast Two-Hybrid Assay of EsMYB9 and bHLH TFs

To analyze the interaction of *EsMYB9* with *bHLH* TFs known to regulate the flavonoid biosynthetic pathway, Y2H assay was performed as previously described by Huang et al. (2016a). The pGADT7 and pGBKT7 vectors (Clontech, Japan) harboring the GAL4 activation domain (AD) and GAL4 DNA-binding domain (BD), respectively, were used for Y2H assay. Firstly, three different regions of EsMYB9 protein, the coding region, MYB domain (aa 1–130) and C-terminal region (aa 131–290) (**Figure 3A**), were inserted into the pGBKT7 vector, generating the EsMYB9-BD, EsMYB9-N-BD and EsMYB9-C-BD constructs for autoactivation activity assay, respectively. For Y2H assay, the full-length coding region of *EsMYB9* was subcloned into the pGADT7 vector, generating the EsMYB9-AD construct, and the EsGL3-BD construct was generated by the full-length coding region of *EsGL3 bHLH* TF (GenBank Accession: KJ010529) inserted into the pGBKT7 vector. All primers used for Y2H constructs were listed in Supplementary Table S1. In addition, the AtTT8-BD and EsTT8-BD constructs which contain the AtTT8 or EsTT8 bHLH protein fused with BD domain had been described previously (Huang et al., 2016b). These plasmid constructs were transformed into yeast strain AH109 using the LiAc/SS carrier DNA/PEG method (Gietz and Schiestl, 2007). Transformed yeast cells were consecutively plated on the double (SD/-Trp/-Leu), quadruple (SD/-Trp/-Leu/-Ade/-His) dropout medium, and quadruple dropout medium supplemented with X-Gal substrate for β-galactosidase assay. The growth of transformed yeast cells was photographed 3–4 days after incubation. Transformed yeast cells harboring pGBKT7-53 + pGADT7-T, pGBKT7-Lam + pGADT7-T construct combinations were selected as a positive and a negative control, respectively.

### Dual-Luciferase Reporter Assay of Transiently Transformed Leaves of *Nicotiana benthamiana*

Transcription activation of *EsMYB9* against promoters of the flavonoid pathway genes from *E. sagittatum* was performed using a dual-luciferase reporter assay in the transiently transformed leaves of *N. benthamiana* as previously described (Huang et al., 2016a). The full-length coding region of *EsMYB9* was transferred from pMD19-T vector into the pGreen II 62-SK vector (Hellens et al., 2005), generating the EsMYB9-containing effector construct. Primers for *EsMYB9* effector construction were listed in Supplementary Table S1. In addition, a total of six reporter constructs containing *EsCHS, EsDFR1, EsDFR2, EsF3H, EsFLS*, or *EsANS* promoters in the pGreen II 0800-LUC reporter vector, and two effector constructs containing *EsTT8* or *AtTT8 bHLH* TFs in the pGreen II 62-SK vector were as previously described by Huang et al. (2016a).

These reporter and effector constructs were introduced into *Agrobacterium tumefaciens* GV3101 strain using a electroporation method. Transformed *Agrobacterium* cells were grown on LB agar supplemented with rifampicin, gentamycin, and kanamycin antibiotics and incubated at 28°C for 2–3 days. The confluent bacteria was scraped and suspended in the infiltration buffer [10 mM MES (pH 5.5), 10 mM MgCl_2_, 150 μM acetosyringone] to an OD_600_ of 0.2–0.3, and then was kept at room temperature without shaking for at least 2–3 h before infiltration. An aliquot of 100 μL of *Agrobacterium* culture transformed with the promoter-containing reporter construct and an aliquot of 450 μL of a second *Agrobacterium* cultures transformed with the MYB and/or bHLH-containing effector constructs were mixed for transient transformation. Approximately 200 μL of this culture mixture was infiltrated into three-four young leaves of each plant, with two-three points for each leaf. At least four-five independent plants were used for each treatment. Finally, the dual-luciferase assay was carried out 3–4 days after inoculation.

A Dual-Luciferase Reporter Assay System kit (Promega, United States) was used to perform the dual-luciferase assay of transiently transformed leaves of *N. benthamiana*, following the method described previously by Huang et al. (2016a). Briefly speaking, the leaf disks with a diameter of 1 cm were collected from the infiltrated points using a puncher, and finely ground in 500 μL of Passive Lysis Buffer. This crude extract was diluted 50-fold with Passive Lysis Buffer and then an aliquot of 10 μL of diluent was taken out and measured in 40 μL of Luciferase Assay Buffer. Another 40 μL of Stop and Glow Buffer was then added and a second luminescence measurement was recorded. The measurement of luminescence unit was carried out using a GloMax 20/20 luminometer (Promega, United States) with a 5 s delay and 10 s integrated measurement. Transcriptional activity data were presented as the ratio of LUC to REN luminescent activity. Only the promoter-LUC reporter constructs (without any TF) were used as the background controls.

## Author Contributions

WH and YW initiated and designed the research. WH performed and analyzed the experiments. HL contributed to materials. WH wrote the paper, and YW revised the paper.

## Conflict of Interest Statement

The authors declare that the research was conducted in the absence of any commercial or financial relationships that could be construed as a potential conflict of interest.

## Supplementary Material

FIGURE S1 Gel image of semi-quantitative RT-PCR assay of *EsMYB9* in transgenic tobacco flowers carrying *EsMYB9* gene. Three MYB9 expressing transgenic tobacco lines and the control plant carrying the empty vector were used for semi-quantitative RT-PCR assay, and tobacco *Actin* gene was used as a reference.

FIGURE S2 Flavonoid content measurement and qPCR assay of the flavonoid-related genes in sucrose-treated leaves of GD1 population of *Epimedium sagittatum*. Plant of GD1 population that contain only epimedin C as the main bioactive components was used as materials for sucrose treatment. The sterilized shoots were cultured in MS basal medium supplemented with 3 or 5% (w/v) of sucrose. The young leaves and petioles were harvested 8 days after treatment for further analysis. The content of epimedin C and total flavonoid **(A)**, the expression levels of flavonoid biosynthetic genes **(B)** and regulatory genes **(C)** by qPCR assay were indicated. Each column represents mean value with SD bar from three replicates. Asterisk indicates a significant difference from the sucrose 3% used as the control (*P* < 0.05, LSD test).
