# Supplementary figures and images for: Functional Characterization of a Novel R2R3-MYB Transcription Factor Modulating the Flavonoid Biosynthetic Pathway from Epimedium sagittatum
Source: Front Plant Sci. 2017 Jul 19;8:1274. doi: 10.3389/fpls.2017.01274 (PMC5515856; doi:10.3389/fpls.2017.01274)

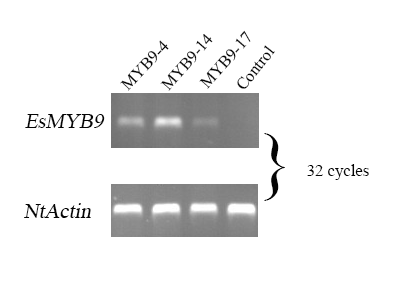

Supplement: FIGURE S1 — Gel image of semi-quantitative RT-PCR assay of EsMYB9 in transgenic tobacco flowers carrying EsMYB9 gene. Three MYB9 expressing transgenic tobacco lines and the control plant carrying the empty vector were used for semi-quantitative RT-PCR assay, and tobacco Actin gene was used as a reference. [file Image_1.TIF]

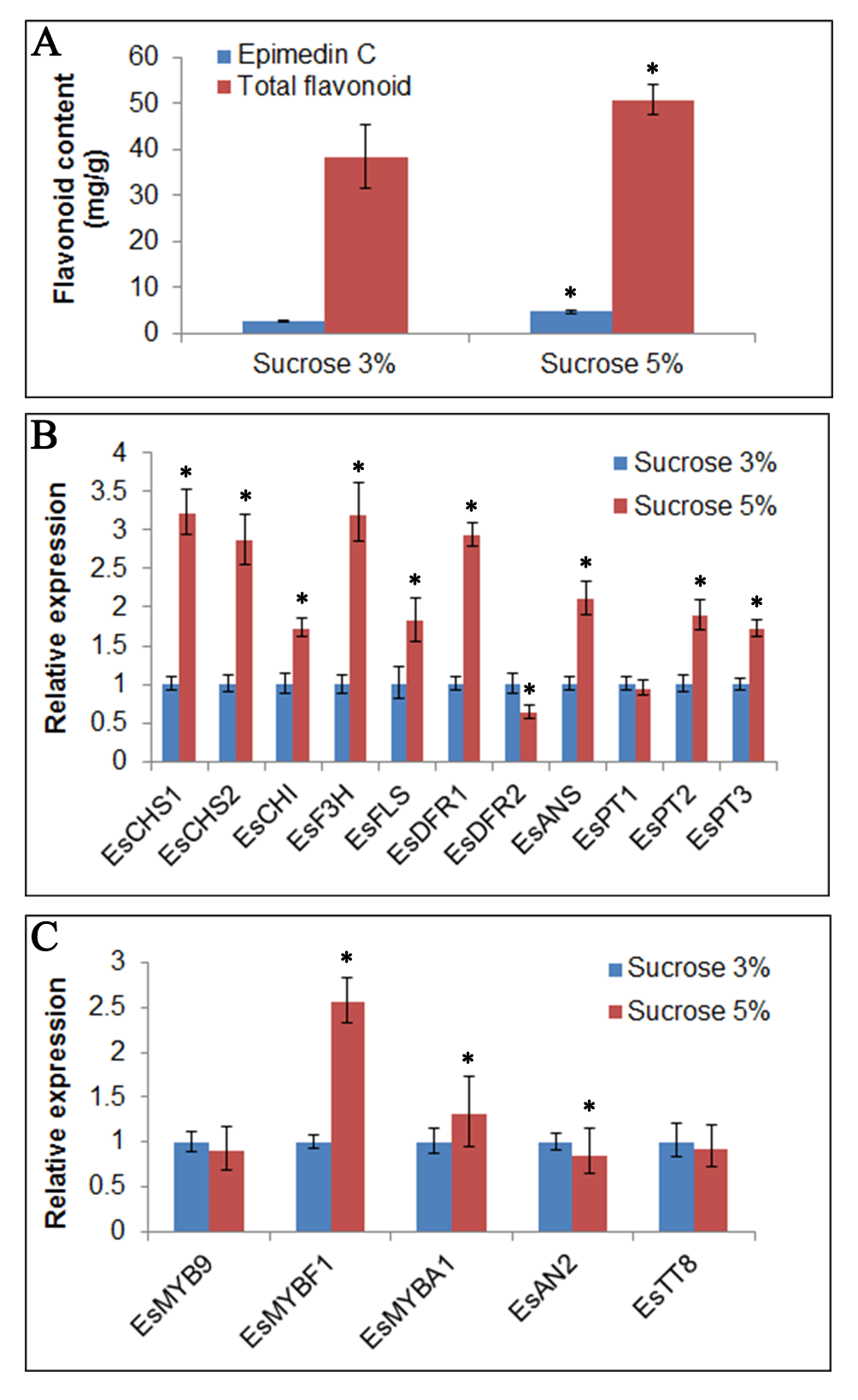

Supplement: FIGURE S2 — Flavonoid content measurement and qPCR assay of the flavonoid-related genes in sucrose-treated leaves of GD1 population of Epimedium sagittatum. Plant of GD1 population that contain only epimedin C as the main bioactive components was used as materials for sucrose treatment. The sterilized shoots were cultured in MS basal medium supplemented with 3 or 5% (w/v) of sucrose. The young leaves and petioles were harvested 8 days after treatment for further analysis. The content of epimedin C and total flavonoid (A), the expression levels of flavonoid biosynthetic genes (B) and regulatory genes (C) by qPCR assay were indicated. Each column represents mean value with SD bar from three replicates. Asterisk indicates a significant difference from the sucrose 3% used as the control (P < 0.05, LSD test). [file Image_2.TIF]
