# Supplementary material for: Functional Characterization of a Novel R2R3-MYB Transcription Factor Modulating the Flavonoid Biosynthetic Pathway from Epimedium sagittatum
Source: Front Plant Sci. 2017 Jul 19;8:1274. doi: 10.3389/fpls.2017.01274 (PMC5515856; doi:10.3389/fpls.2017.01274)
Supplement: Supplementary file 3 [file Table_1.DOCX]

| **Primers for function** | **Forward sequence (5’-3’)** | **Reverse sequence (5’-3’)** |
| --- | --- | --- |
| Full-length cDNA cloning for EsMYB9 overexpression construct | CTGCCGTGCTGTGTAACATAGA | TCAATGTAACCTATGACAACAAGAAGC |
| Full-length cDNA cloning for EsTT8 overexpression construct | TAATTGGGTTCCGGTAGAAAGAAT | TACAGTCAATCACATTATATACCAACACT |
| EsMYB9 primers for qPCR assay | ATACCAACAACCCAAACC | CTTAGCAATGACTCCAGAAC |
| EsTT8 primers for qPCR assay | GTGGCTACTCAAGTACATTC | GATTCGCACTCAACTCATC |
| EsMYB9 N-terminus for yeast two-hybrid (BD) | GCCATATGAGAAACCCTAGCACTGGAG | CGGTCGACTAAGTGGGTGTTCCAGTAATTCT |
| EsMYB9 C-terminus for yeast two-hybrid (BD) | GCCATATGAGCAAGAAACTCATCAGCCAAG | CGGTCGACAGAACCTTTCTCGGCATGATC |
| EsMYB9 ORF for yeast two-hybrid (BD) | GCCATATGAGAAACCCTAGCACTGGAG | CGGTCGACAGAACCTTTCTCGGCATGATC |
| EsMYB9 ORF for yeast two-hybrid (AD) | GCCATATGAGAAACCCTAGCACTGGAG | GGCTCGAGAGAACCTTTCTCGGCATGATC |
| EsGL3 ORF for yeast two-hybrid (BD) | GCGAATTCATGGCAGCTGAAGCTCACAAC | GGGTCGACGGTCGTGTCAATAATTGTTAAAATG |
| EsMYB9 ORF for dual-luciferase assay | CGGGATCCATGAGAAACCCTAGCACTGGAG | CGGTCGACTCAAGAACCTTTCTCGGCATG |

**Table S1. List of primers used for functional characterization of *EsMYB9* transcription factor from *Epimedium sagittatum***

Note: Underlines represent enzyme recognition sites

**Table S2. List of primers used for quantitative PCR assay of the flavonoid pathway genes in transgenic tobacco**

| Gene name | Accession number | Forward primer (5’-3’) | Reverse primer (5’-3’) | PCR length |
| --- | --- | --- | --- | --- |
| *NtPAL* | X78269 | CGATAGACTTGAGGCATTTG | AGGTTCTCTTAGCGACTTG | 78 |
| *NtC4H* | AB236952 | GTTGCCTCCTCCAGGACAGTC | TGGTGGAATGCTTCAAAATGTG | 83 |
| *Nt4CL* | U50845 | GCGACATTGGGTTCATTG | TTCTCCTGCTTGCTCATC | 176 |
| *NtCHS* | AF311783 | AGCGAGCATAAGGTTGAG | ACCACCACTATGTCTTGTC | 164 |
| *NtCHI* | AB213651 | CTTTTCTCGCCGCTAAATG | TTTCTGCCACCTTCTCTG | 159 |
| *NtF3H* | AB289450 | GAGGCAATGGGCTTAGAG | TCAGTGTGTCGTTTCAGTC | 128 |
| *NtF3'H* | AB289449 | AGCCATAGTCAAGGAAACC | CTCACAACTCTCGGATGC | 79 |
| *NtDFR* | EF421429 | TAAGAAGATGACAGGATGGATG | TGGCGGTATGATGCTAATG | 109 |
| *NtFLS* | DQ435530 | GTCCCATATAACCATTCTTGTC | CACTCTTGTATTTCCCATTGC | 152 |
| *NtANS* | AB289447 | CTACATTCCAGCAACAAGTG | GTCCCAGCCCAATAGAAAG | 86 |
| *NtUF3GT* | GQ395697 | TCCTTCTTCAACACTTCACAATCC | AATAGGGGTAGTGCCATTTGTTTC | 120 |
| *NtAn1a* | HQ589208 | ACCATTCTCGAACACCGAAG | TGCTAGGGCACAATGTGAAG | 97 |
| *NtAn1b* | HQ589209 | CTTGAACACTTCTCAAACCGA | TGCTAGGGCACAATGTGAAG | 100 |
| *NtTub1* | AJ421411 | TCCGTGGTGATGTTGTG | TGGTGGCTGATAGTTGATAC | 125 |
